# Nanomaterials Induced Genotoxicity in Plant: Methods and Strategies

**DOI:** 10.3390/nano12101658

**Published:** 2022-05-12

**Authors:** Marta Marmiroli, Nelson Marmiroli, Luca Pagano

**Affiliations:** 1 Department of Chemistry, Life Sciences and Environmental Sustainability, University of Parma, Parco Area delle Scienze 11/A, 43124 Parma, Italy; 2 Consorzio Interuniversitario Nazionale per le Scienze Ambientali (CINSA), Parco Area delle Scienze 11/A, 43124 Parma, Italy; nelson.marmiroli@unipr.it

**Keywords:** nanomaterials, plant genotoxicity, methods, biomarkers, organelles

## Abstract

In recent years, plant-nanomaterial interactions have been studied, highlighting their effects at physiological and molecular levels. Transcriptomics and proteomics studies have shown pathways and targets of nanomaterial exposure and plant response, with particular regard to abiotic stress and oxidative stress. Only little information has been reported on engineered nanomaterial (ENMs) interactions with plant genetic material, both at a genomic and organellar DNAs level. Plants can be useful experimental material, considering they both contain chloroplast and mitochondrial DNAs and several plant genomes have been completely sequenced (e.g., *Arabidopsis thaliana*, *Solanum lycoperiscum*, *Allium cepa*, *Zea mays*, etc.). In this mini review, the methods and the evidence reported in the present literature concerning the level of genotoxicity induced by ENMs exposure have been considered. Consolidated and potential strategies, which can be applied to assess the nanomaterial genotoxicity in plants, are reviewed.

## 1. ENM Genotoxicity in Plant: The Current State

The global market for nanotechnology might grow from USD 5.2 bln in 2021 to USD 23.6 bln by 2026, with annual growth rate (CAGR) of 35.5%, respectively for the years 2021–2026. The North American market for nanotechnology is estimated to grow from USD 1.6 bln in 2021 to USD 7.2 bln by 2026, at a CAGR of 34.5% for the period 2021–2026, while the Asia–Pacific market for nanotechnology is estimated to grow from USD 1.2 to USD 6.0 bln, at a CAGR of 37.6%, respectively, for the same time period, as reported by Nanotechnology Services Global Market Report 2022.

Nanotechnology has captured the attention of a wide range of industries in many sectors, gaining in a short period large attraction and significant public investments in research and development, in addition to increasing private-sector investments. Many governments are implementing the application of nanotechnology notwithstanding the associated risks and uncertainties [1]. Nanotechnology allows the development and improvement of completely new products, processes, and services [2].

However, engineered nanomaterials (ENMs) are in the process of being dispersed into the environment, coming into contact with non-mammal organisms and plants [3,4]. So far, scientists have just started to investigate the impact of nanomaterials on plants, which has contrasting outcomes depending on the type of nanomaterial and on the plant species [5]. The field of nanotoxicology has been extended from microorganisms to plants and animals, even if the idea of ENM genotoxicology for plants is not so widespread. In fact, a search in Scopus [6] for publications with the word “Nanotoxicology” since 2013 produced 625 results. Research in the same timeframe, from 2013 to 2022, using the word “nanomaterial genotoxicology” produced only four outcomes. A more extensive database research has been conducted by Ghosh et al. [7], who found that there are few papers dealing with the genotoxicity of the nanoparticles in respect to other effects that nanoparticles exert on plants.

In the field of toxicology, the term genotoxicity generally refers to any kind of damage to the genetic material, the genome, as cytotoxicity indicates injury to the cell instead. Toxic effects to the genetic material have attracted great attention for many reasons, including in particular that the genome of germ cells, the reproductive cells, determine all heritable characteristics of organisms [8]. Investigation of injury to the genome has led to the definition of a specific kind of toxicity, genotoxicity, and to the development of the subspecialty of genetic toxicology [9].

Several plant species have the intrinsic capability of being used as multiple genetic assay systems. These plant genetic systems have played important roles in detecting new mutagens and developing techniques later used in other systems for advancing mutagenesis knowledge. Some of the mainly used higher plant species are: *Allium cepa* L. (2n = 16), *Arabidopsis thaliana* L. (2n = 10), *Crepis capsularis* (L.) Wallr (2n = 6), *Glycine max* L. (2n = 40), *Hordeum vulgare* L. (2n = 14), *Solanum lycopersocum* L. (2n = 16), *Nicotiana tabacum* L. (2n = 48), *Pisum sativum* L. (2n = 14), *Tradescantia* Ruppius ex L. (2n = 24), *Vicia faba* L. (2n = 12) and *Zea mays* L. (2n = 20) [10].

## 2. Mechanisms of ENM-Induced Genotoxicity

In vitro and in vivo characterization of the response to ENM exposure in both growth media and biological matrices have been extensively discussed in recent years [11]: uptake, pathways, biotransformation, and the mechanisms of ENM genotoxicity. In vitro and in vivo characterization of the response to ENM exposure in both growth media and biological matrices have been extensively discussed in recent years [11]: uptake, pathways, biotransformation, and the mechanisms of ENM genotoxicity. Different mechanisms can be exploited depending on the different ENM physico-chemical properties: (i) ENMs simply able to pass through the cellular membrane lipid bilayer, depending on several factors such as size, charge, hydrophobicity, composition and shape; (ii) endocytosis processes by which ENMs are taken up and accumulated in plant tissues, as well as Trojan horse mechanism and possible biotransformation processes (including corona protein interactions), lead to ENMs accumulation in plant cells; (iii) the utilization of membrane transporters which can mediate the translocation of ENMs into the plant cell, due to their affinity to the transporter itself [12,13]. As a result, ENMs response can be explicated by two different mechanisms: effects directly ascribed to the ENMs interaction with the cellular components, or its biotransformed physico-chemical forms (including ions released, depending on the ENM stability) [14] and indirectly, due to ROS production, increase mediated by mitochondrion and chloroplast functionality alteration, leading to a general cellular oxidative stress increase by triggering ENM-induced cytotoxicity and genotoxicity mechanisms [12]. The response observed is an effect of the activation defense mechanisms, including antioxidant defense mechanisms, apoptosis and secondary metabolite (e.g., phytohormone) production and antioxidant enzymes [11].

As a key metabolite, ROS are necessary in plants for many important signaling reactions, however they also constitute by-products in aerobic metabolism that can induce oxidative damage in plants [15]. It has been demonstrated that nanoparticles and ROS can directly enter the nucleus of the plant cell and, by binding chromatin and/or interacting with DNA, induce damages [16], showing potential mutagenic effect.

For nanoparticles (NPs) such as Ag NPs (coated and uncoated), carbon nanotubes, ZnO NPs, Al_2_O_3_ NPs, Fe_2_O_3_ NPs, Co_3_O_4_ NPs, and NiO NPs, the main features that determine genotoxicity have been found to be ions release, dimension, and zeta potentials [7]. As a fact, these features contribute to the penetration of the nanoparticle into the cell nucleus and the consequent damage to DNA [17]. Several assays have been developed that use higher plants to measure the mutagenic effects of chemicals in general as indicators of carcinogenicity. These assays using plants require less complex equipment and materials than many other genotoxicity tests, which is a potential advantage, particularly when research resources are limited [18]. Standard genotoxicity tests have been reviewed by the Gene-Tox program of the U.S. Environmental Protection Agency (EPA) concerning gene mutation, chromosomal effects and DNA damage repair on the following plants: *A. thaliana*, *G. max*, *H. vulgare*, *Tradescantia*, *Z. mays* [18,19]. Early studies on plants progressed to more sophisticated and complex assays on many other plants, and to many more materials including ENMs [7,17].

In this minireview, the most important genotoxicity assays applied on plants are explained, with a focus on how they can be utilized to determine the genotoxic effects for nanoparticles, which include standard techniques available and new tools and instruments. DNA damage may cause epigenetic changes, through covalent DNA modification, histones modification, and regulation of non-coding RNAs (miRNAs, lncRNAs, piRNAs). Modifications at the level of DNA methylation (global or gene-specific) may have a profound impact on chromatin remodeling and on locus-specific gene expression, respectively [20].

## 3. Current Methods and Functional Applied Strategies

### 3.1. Standard Techniques

**Figure 1:**
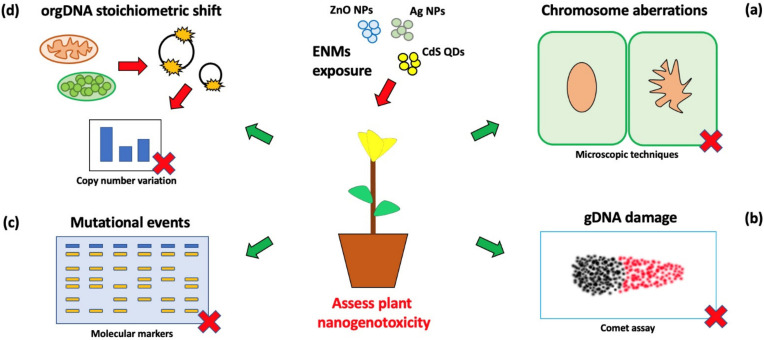
Schematic representation of the methodologies utilized to highlight plant ENM genotoxicity: (**a**) microscopic techniques to highlight chromosomal aberrations, (**b**) electrophoresis-based methods (e.g., comet assay) to highlight genomic DNA (gDNA) damage, (**c**) molecular markers (e.g., RAPD) to show mutational events and (**d**) Real time PCR based methods to highlight copy number variation (stoichiometric or sub-stoichiometric shift) in plastid (ptDNA) or mitochondrial (mtDNA) genomes. These techniques can be utilized as Alternative Testing Strategies (ATS), in assessing and/or characterizing the risk associated with ENMs exposure/effects, not only in experimental controlled conditions, but also in monitoring of realistic scenarios, at early exposure stages.

From an operational point of view, different approaches can be utilized to pinpoint the genotoxic effects of ENMs on plant DNA [7]. All these approaches are able to assess ENM genotoxicity from different points of view, showing potential advantages and disadvantages in terms of sensitivity and resolution, respectively. Methods described and relevant examples are schematized in Figure 1.

Among the major effects observed from the exogenous genotoxic effects on plant genomes, the chromosomal aberrations, which are the result of structural and numerical chromosome changes, preferentially within heterochromatic regions, are composed mainly of repetitive DNA sequences [21]. Optical, fluorescence and confocal laser scanning microscopy techniques are able to highlight aberrations at the level of the chromosome structure, including chromosomal breaks, sticky, multipolar, and laggard chromosomes, as well as micronucleus formation [22,23,24].

Chromosomal aberrations have been observed by Pakrashi et al. [25], studying the effect of titania nanoparticles (TiO_2_ NPs) on *Allium cepa* L. root tips, in the range 0–100 mg L^−1^. Optical and fluorescence microscopic analyses showed a dose-dependent frequency of the aberration appearance, which includes chromosomal breaks, chromosome stickiness during metaphase, multiple micronucleus formation, as well as the occurrence of binucleate cells. Confocal microscopic images highlighted the formation of chromosomal bridges, in addition to a distorted and notched nucleus.

Similarly, Panda et al. [26] observed micronucleus mitotic aberrations formations in *Allium cepa* L. cells exposed to 0–80 mg L^−1^ of different forms of silver ionic colloidal nanoparticles (Ag NPs). Additionally, in this case, the percentage of increase in aberrations was concentration dependent.

Silva and Monteiro [27] investigated the genotoxic and phytotoxic impacts of silica-based nanomaterials (SiO_2_ NPs, in a range between 0.54–1.82 g L^−1^) using root tip cells of *Allium cepa* L., highlighting chromosomal aberrations and delays in mitosis due to disturbed metaphase. Sun et al. [28] studied the cytotoxic and genotoxic effects of ZnO NPs (5–50 mg L^−1^) in root meristems of *Allium cepa* L. cells by cell membrane integrity, metabolic activity, reactive oxygen species (ROS) accumulation, DNA damage and chromosomal aberration, highlighting how ZnO NP accumulation within cell nucleuses affected cell mitosis, inducing chromosome breaks, bridges, stickiness, and micronuclei formation. As often reported, the utilization of *Allium cepa* L. is considered an efficient bioindicator in genotoxicity testing, due to its reduced number of chromosomes and rapid root growth rate [29]. Abdelsalam et al. [30] investigated the effects of foliar application of (nitrogen-phosphorus-potassium) NPK nanoparticles (2.5 to 5 kg ha^−1^) for two harvest seasons on *Triticum aestivum* L. as an alternative to conventional fertilizers, assessing yield and genotoxic effects. Although fertilization with NPK nanoparticles produced an increase in yield, root-tip cells showed various types of chromosomal aberrations such as multinuclei, micronuclei, chromosome deletion, lagging chromosome and cell membrane damage, and the NPK nanoparticles treatment at 5 kg ha^−1^ produced 35.7–38.9% of abnormal cells. With a similar approach, Abdelsalam et al. [31] tested on *Triticum aestivum* L. seeds the utilization of (amino-zinc) AZ nanoparticles (50–150 mg L^−1^) on in vitro medium for 8, 16, or 24 h. Genotoxicity was evaluated in root meristems, revealing mitotic activity variations, chromosomal aberrations, and micronuclei formation and a growth inhibit of the normal cellular function.

### 3.2. Gel Electrophoresis-Based Methods

DNA damage in individual plant cells can be highlighted by gel electrophoresis-based methods [32]: cells embedded in agarose on a microscope slide are lysed with detergent and high salt concentrations to form nucleoids containing supercoiled loops of DNA linked to the nuclear matrix; subsequent electrophoresis conducted at high pH produces structures resembling comets, which can be observed by fluorescence microscopy. The intensity of the “comet tail” reflects the breaks in DNA sequences. Comet assay is able to detect DNA single-strand breaks, DNA double-strand breaks, and the formation of apoptotic nuclei [33]. This assay is often utilized as a confirmation method for microscopic evidence [26,34,35].

Several examples can be found in recent literature related to ENM genotoxicity in plants: Panda et al. [26], through comet assay, observed a significant DNA damage rate determined by dose-dependent Ag NPs exposure and correlated to ROS formation. Faisal et al. [36] utilized the comet assay to assess the genotoxic effects in *Solanum lycopersicum* L. seedlings exposed to NiO NPs (0–2 g L^−1^). Analyses showed a significant increase in genomic DNA damage, and an increase in the number of apoptotic (21.8%) and necrotic (24.0%) cells. Ciğerci et al. [34] studied Indium tin oxide (ITO, In_2_O_3_/SnO2, ration 90/10%) particles (1–100 mg L^−1^), observing a significant increase in DNA damages in *A. cepa* root meristematic cells, highlighting potential alterations in the cell cycle, as demonstrated by the higher number of cells able to enter into mitosis, as compared with the untreated controls. Thiruvengadam et al. [37] studied physiological, metabolic, and transcriptional effects of Ag NPs (1–10 mg L^−1^) *Brassica rapa* spp. observing a dose dependent DNA damage effects in turnip cells. Sun et al. [28] confirmed by comet assay the chromosomal aberration generated in *A. cepa*, highlighting a significant increase in DNA fragmentation after ZnO NPs exposure.

### 3.3. Molecular Markers and Biomarker Assays

Not only electrophoresis-based methods and chromosomal aberration analyses are utilized to detect potential genotoxic effects. Molecular markers can be also implemented as tools to detect the ENMs effect on genetic materials [38]. Molecular markers are defined as fragments or amplicons of DNA associated with a certain location within the genome. Molecular markers can be used as a biotechnological tool to identify and characterize a particular sequence of DNA when there is a limited knowledge of the sequence itself. This is the case, for example, of Random Amplified Polymorphic DNA (RAPD), markers based on PCR amplification of DNA fragments from random segments of genomic DNA, with a single primer of an arbitrary nucleotide sequence [39]. RAPDs do not require specific knowledge of the DNA sequence of the target organism. The occurrence of mutation at the level of DNA, particularly at the site that was previously complementary to the primer, will not allow amplicon production, resulting in a different pattern of amplified DNA fragments, which results in a molecular marker that is mainly dominant [40]. Since the early 1990s, several molecular marker tools have been developed in order to increase the detail of the physical genomic mapping and QTL analysis, with pros and cons related to the intrinsic properties of each molecular marker, respectively [41].

Molecular markers can be also utilized as tools to determine potential mutations at the level of the DNA sequence [42], which can support or validate data previously obtained, but also to isolate potential targets functional to biomarker characterization and development [43,44].

Hosseinpour et al. [45] studied the effects of the application of ZnO NPs (0–40 mg L^−1^) and plant growth promoting bacteria on *S. lycopersicum* L. under salt stress, with particular regard to DNA damage and cytosine methylation changes. RAPD analysis has been performed to determine the effects of co-exposure to bacteria and ZnO NPs on tomato genomic DNA. The rate of polymorphism observed in case of salinity stress treatment (42.2%) was a decrease in case of exposure to ZnO NPs and/or plant growth promoting bacteria from 32.4% to 25.3%, respectively. The results obtained through the application of different bacteria and ZnO NPs concentrations suggest the inverse relationship between the level of cytosine methylation and salinity stress tolerance. Mosa et al. [46] studied the genotoxic effects and genomic alterations in *Cucumis sativus* L. of copper-based nanoparticles (Cu NPs) using the RAPD technique. Cu NPs (0–200 mg L^−1^) showed a concentration-dependent increase rate of polymorphism occurrence, highlighting the Cu NPs genotoxic effect. Kokina et al. [47] studied the impact of iron oxide nanoparticles (Fe_3_O_4_ NPs, 0–4 mg L^−1^) on *Medicago falcata* L. The utilization, in this case, of the RAPD technique highlighted the genotoxic effect of Fe_3_O_4_ NPs, which induced genomic DNA modifications. This type of PCR-based molecular marker for its randomic amplification nature may be subject to experimental or technical variability, and thus requires procedures of validation [38,39]. Several other type of molecular markers and biomarkers can be utilized as more reliable tools to assess genomic variations, either at the level of genomic DNA (gDNA) and plastid and mitochondrial DNA (ptDNA, mtDNA). Pagano et al. [44] highlighted a modulation of the organellar functionality in *Arabidopsis thaliana* L. Heynh in direct comparison to a modulated organelle genome replication level, upon exposure to CeO_2_ NPs, FeOx NPs, ZnS QDs, CdS QDs (80–500 mg L^−1^). In this case, multiple target genes at the level of ptDNA and mtDNA were utilized as structural markers to assess the potential variations at the level of DNA replication by real time qPCR. In particular, CdS QD exposure triggered potential variations at the sub-stoichiometric level of the two organellar genomes, while nanoscale FeOx NPs and ZnS QDs exposure triggered an increase in organellar DNA copy numbers. These findings suggested how modification in organellar genomes stoichiometry may result from a potential morpho-functional adaptive response to ENM exposure, which led to decreased rates of photosynthesis and cellular respiration.

### 3.4. Other Approaches

Other approaches, which included *A. thaliana* transgenic lines for homologous recombination and transcriptional gene silencing, were adopted to assess the genotoxicity of ZnO NPs [48]. The results showed, at the level of roots, how exposure to ZnO NPs (0–20 mg L^−1^) resulted in an increase in homologous recombination (in particular the gene *atRad54-GFP-GUS* expression) and a reduction in transcriptional gene silencing in leaves (which contained the multicopy construct *P35S::GUS*), which can be ascribed to genotoxic effects triggered by ZnO NPs dissolution to free Zn ions. Methods described and relevant examples are reported in Table 1.

## 4. Conclusions

In conclusion, in recent years, different techniques, previously exploited for animal cells, have been developed and applied to plants to assess the genotoxic effects related to ENM exposure. These approaches, considering their properties, and the relative pros and cons, which include high/low resolution vs. high/low target specificity, may be implemented for cross-validation of the results obtained. This may also include potential applications related to the utilization of novel methods of mutagenesis (e.g., CRISPR-*Cas9*) [49].

In this context, plants and microorganisms can be utilized as model organisms instead of animal models for Alternative Testing Strategies (ATS) to assess and characterize the risk, with particular regard to genotoxicity, related to ENMs exposure/effects [44,50]. Adoption of ATS for new organisms, endpoints, and span of variations in experimental scale and complexity have been increasingly functional in nanotoxicological literature through iterative processes able to combine results from physiological and molecular approaches [51]. Moreover, the monitoring of ENMs dispersal in the environment, especially at very early exposure stages and in realistic scenarios, can be further implemented [52] in accordance with the recently published EFSA guidance on risk assessment of the application of nanoscience and nanotechnologies in the food and feed chain, human and animal health, which considers in vitro/in vivo toxicological testing (e.g., in vitro degradation, toxicokinetics, genotoxicity, local and systemic toxicity), and the European Registration, Evaluation Authorization and Restriction of Chemicals (REACH) protocols for chemical safety assessment [1,53].

## Figures and Tables

**Table 1:** Reference list of relevant experiments performed with different tools to identify ENM genotoxic effects in plants.

ENM	Treatment ^(*)^	Plant	Analyses	Reference
TiO_2_ NPs	Conc.: 0–100 mg L^−1^ (hydroponic), 4 h treatment	*Allium cepa* L.	Chromosome aberration	Pakrashi et al. [25]
Ag NPs	Conc.: 0–80 mg L^−1^ (hydroponic), 1 h treatment	*Allium cepa* L.	Chromosome aberration, Comet assay	Panda et al. [26]
SiO_2_ NPs	Conc.: 0.54–1.82 g L^−1^ (hydroponic), 24 h treatment	*Allium cepa* L.	Chromosome aberration	Silva and Monteiro [27]
ZnO NPs	Conc.: 5–50 mg L^−1^ (hydroponic), 36 h treatment	*Allium cepa* L.	Chromosome aberration, Comet assay	Sun et al. [28]
NPK particles	Conc: 2.5–5.0 kg ha^−1^ (in soil, foliar spray), two harvest seasons	*Triticum aestivum* L.	Chromosome aberration	Abdelsalam et al. [30]
AZ particles	Conc: 50–150 mg L^−1^ (in vitro), 8, 16, 24 h treatment	*Triticum aestivum* L.	Chromosome aberration	Abdelsalam et al. [31]
NiO NPs	Conc.: 0–2 g L^−1^ (in vitro), 12 d treatment	*Solanum lycopersicum* L.	Comet assay	Faisal et al. [36]
In_2_O_3_/SnO_2_ particles	Conc.: 1–100 mg L^−1^ (hydroponic), 4 h treatment	*Allium cepa* L.	Comet assay	Ciğerci et al. [34]
Ag NPs	Conc.: 1–10 mg L^−1^ (in vitro), 14 d treatment	*Brassica rapa* spp.	Comet assay	Thiruvengadam et al. [37]
ZnO NPs	Conc.: 0–40 mg L^−1^ (in vitro), 14 d treatment	*Solanum lycopersicum* L.	RAPD	Hosseinpour et al. [45]
Cu NPs	Conc.: 0–200 mg L^−1^ (in vitro), 21 d treatment	*Cucumis sativus* L.	RAPD	Mosa et al. [46]
Fe_3_O_4_ NPs	Conc.: 0–4 mg L^−1^ (hydroponic), 35 d treatment	*Medicago falcata* L.	RAPD	Kokina et al. [47]
CeO_2_ NPs, FeOx NPs, ZnS QDs, CdS QDs	Conc.: 80 mg L^−1^ (CdS QDs), 500 mg L^−1^ (CeO_2_ NPs, FeOx NPs, ZnS QDs), (in vitro) 21 d treatment	*Arabidopsis thaliana L.*	mtDNA, ptDNA copy number variation	Pagano et al. [44]
ZnO NPs	Conc.: 0–20 mg L^−1^ (hydroponic), 20 d treatment	*Arabidopsis thaliana L.*	Gene silencing	Yang et al. [48]

*, treatment conditions information includes concentration, experimental setup, and time of exposure utilized. Reference list order in the table reflects the order of appearance in the text, depending on the type of analyses performed.

## Data Availability

The data presented in this study are available on request from the corresponding authors.
